# An acute phase reaction with intravenous bisphosphonate use in a patient with recently diagnosed Graves’ disease

**DOI:** 10.1002/ccr3.1022

**Published:** 2017-06-19

**Authors:** Katharine Gupta, Jane Estrella, Rohit Rajagopal, Praseetha Shanmugalingam, David Simmons

**Affiliations:** ^1^ Macarthur Diabetes and Endocrine Centre Campbelltown Hospital Campbelltown New South Wales Australia; ^2^ Department of Medicine Campbelltown Clinical School Western Sydney University Campbelltown New South Wales Australia

**Keywords:** Bisphosphonate, endocrinology, Graves’, hypercalcemia, hyperthyroidism

## Abstract

Given the immune background, we hypothesize that active Grave's hyperthyroidism is a risk factor for an acute phase reaction associated with the use of bisphosphonates. We recommend that in patients with Graves’ thyrotoxicosis and hypercalcemia, consider the risk of an acute phase reaction if planning to give bisphosphonate therapy.

## Introduction

Intravenous bisphosphonates are usually well tolerated. An acute phase reaction occasionally develops and several risk factors have already been identified 1. We report a patient with Graves’ hyperthyroidism who developed an acute phase reaction following intravenous bisphosphonates. We hypothesize that this reaction was secondary to immunologic priming by the Graves’.

## Clinical Record

A 22‐year‐old female presented with headache, neck pain, and photophobia. The initial impression was viral meningitis and this was excluded after cerebrospinal fluid examination was noted to be normal. Incidentally, her calcium level was found to be elevated at 3.07 mmol/L. The neurologic team subsequently tested her thyroid function tests and biochemistry revealed Grave's disease and PTH‐independent hypercalcemia (Table 1). Her care was taken over by the endocrinology team.

**Table 1:** Investigations for PTH‐independent hypercalcemia

TSH	<0.02 mIU/L (0.40–4.20 mIU/L)
Free T4	92.2 pmol/L (11.0–22.0 pmol/L)
T3	42.4 pmol/L (3.0–6.2 pmol/L)
TRAB	33 IU/L
Corrected Calcium	3.25 mmol/L (2.10–2.60 mmol/L)
PTH	0.2 pmol/L (1.5–7.0 pmol/L)
25, hydroxyl vitamin D	93 nmol/L (>75 nmol/L)
1,25 dihydroxy vitamin D	18 pmol/L (60–200 pmol/L)
Chest X‐ray	Normal hilum
Serum ACE	40 IU/L (8–53 IU/L)
Myeloma Screen	No paraprotein in both serum and urine
PTHrP	Undetectable

Further history revealed that she had been experiencing a 1‐month history of severe headache, vomiting, nausea, anorexia, poor sleep, hot flushes, and a 10 kg weight loss and important negatives were that she had no fever, no preceding physical or psychological trauma, and no risk factors for autoimmune thyroid disease other than her young age and female sex (no personal history or family history of thyroid or autoimmune diseases, no exposure to iodine or iodine contrast, not currently pregnant and no recent child birth, never smoked, and no illicit drug use). She had no previous medical history, her only regular medication was Depo‐Provera and she had no known allergies.

Physical examination showed normal mentation and vital signs were tachycardia (120 beats per minute), a blood pressure of 130/70 mmHg, a respiratory rate of 16 breaths per minute, oxygen saturations of 100% on room air, and a temperature of 37.2°C. She also had a mild peripheral tremor, and a large, diffuse, smooth, non‐tender goiter with a faint bruit. Otherwise, cardiorespiratory, abdominal, and neurologic examinations were normal. Her electrocardiograph showed sinus tachycardia. Biochemistry at this time was normal (Table 2).

**Table 2:** Investigations on admission

Sodium	143 mmol/L (135–145 mmol/L)
Potassium	4.1 mmol/L (3.5–5.2 mmol/L)
Urea	3.7 mmol/L (3.0–8.0 mmol/L)
Creatinine	43 *μ*mol/L (45–90 *μ*mol/L)
eGFR	>90 mL/min/1.73 m^2^ (>60 mL/min/1.73 m^2^)
Albumin	36 g/L (38–48 g/L)
Bilirubin	12 *μ*mol/L (<21 *μ*mol/L)
ALP	117 U/L (30–130 U/L)
GGT	16 U/L (<35 U/L)
ALT	22 U/L (5–55 U/L)
AST	21 U/L (5–55 U/L)
Hb	111 g/L (120–150 g/L)
WCC	5.5 × 10^9^/L (4.0–10.0 10^9^/L)
Platelets	222 × 10^9^/L (150–400 10^9^/L)
ESR	27 mm/h (0–12 mm/h)
CRP	1.6 mg/L (<5 mg/L)

The patient was managed on the ward and commenced on metoprolol 25 mg twice daily and carbimazole 10 mg fourth hourly, with subsequent improvement in thyroid function tests (TFTs) (Table 3). Despite improvement in the TFTs, ongoing intravenous fluid therapy (total of 13 L of fluid over 5 days) and loop diuretics (120 mg given on both days 1 and 2 of IV fluid therapy), the patient remained symptomatic and her calcium level remained greater than 3.0 mmol/L (Corr Ca: 3.04 mmol/L, ionized Ca: 1.50 mmol/L). A decision was made to administer 60 mg of intravenous pamidronate over 4 h. This worked well to lower her calcium and subsequently resulted in hypocalcaemia at which time she was commenced on calcitriol. She also required intravenous magnesium replacement.

**Table 3:** Thyroid function tests throughout admission

	TSH (0.40–4.20 mIU/L)	T4 (11.0–22.0 pmol/L)	T3 (3.0–6.2 pmol/L)
Admission	<0.02	92.2 pmol/L	42.4 pmol/L
Pamidronate given	<0.02	55.5 pmol/L	18.8 pmol/L
Carbimazole changed to 10 mg q8 hrly	<0.02	33.5 pmol/L	5.3 pmol/L
Carbimazole ceased	<0.02	25.3 pmol/L	7.3 pmol/L
Carbimazole recommenced	<0.02	36.0 pmol/L	26.7 pmol/L
Discharged	<0.02	21.0 pmol/L	10.3 pmol/L

Eighteen hours following intravenous pamidronate and day 3 of carbimazole therapy, the patient developed recurrent high‐grade temperatures (40°C), rigors, myalgias, epigastric pain, back pain over her lumbar puncture site, pain in both her legs, worsening severity of her headaches, and persistent marked sinus tachycardia (up to 155 bpm). On examination, she looked very unwell, was having rigors, was tender in the epigastrium (positive percussion tenderness), and very tender over her lumbar spine. There was no focal neurologic deficit and no signs of meningism. She was commenced on empirical intravenous Ceftriaxone 1 g daily. This was later ceased after multiple investigations to localize a source of pyrexia were non‐contributory (Table 4). At this time, carbimazole therapy was reduced to 10 mg three times daily (see Table 3 for TFTs).

**Table 4:** Investigations for sepsis

5x blood cultures	No growth
Urine culture	No growth
Stool sample	No clostridium difficile toxin, no bacteria, no cysts, no ova, no parasites
Nasopharyngeal swap	No respiratory viruses on PCR
V/Q scan	No pulmonary emboli
Bilateral lower limb Dopplers	No deep vein thrombosis
MRI spine	No epidural abscess
CT abdomen without contrast	No abnormality

Biochemistry showed elevated serum transaminases (ALT 107 IU/L, AST 119 IU/L) and pancytopenia (WCC 3.1 10^9^/L, Hb 93 g/L, Plts 106 10^9^/L). An autoimmune screen (antinuclear antibody, antineutrophil cytoplasmic antibodies, double‐stranded deoxyribonucleotide acid antibody [DNA], extractable nuclear antigen antibodies, and anti‐liver‐kidney microsomal antibodies) was negative. EBV serology suggested prior infection but no acute infection. CMV, Hepatitis A, Hepatitis B, Hepatitis C, and HIV were all negative. Ultrasound of the abdomen showed mild fatty liver infiltration, but no focal hepatitis or biliary dilatation, no cholelithiasis, or features of acute cholecystitis. The gastroenterology team was consulted and they felt the raised serum transaminases may have been related to the febrile illness or to the carbimazole therapy.

Despite hemoglobin and platelets improvement, the neutropenia worsened (0.5 10^9^/L). Carbimazole was temporarily ceased due to possible carbimazole‐induced agranulocytosis (see Table 3 for TFTs). Counts improved within 12 h of carbimazole cessation and remained normal following re‐introduction of carbimazole 10 mg twice daily (see Table 3 for TFTs). Ibuprofen 400 mg q8 hourly, in addition to paracetamol 1 g q6 hourly, was started with lysis of fever and marked clinical improvement within 12 h. All of her symptoms gradually improved and at 4 days after her first fever, all of her symptoms had resolved. Her liver function tests also continued to improve although they were still deranged on discharge (ALP 122 U/L, GGT 38 U/L, ALT 100 U/L, and AST 41 U/L). On discharge, her calcium was mildly low (2.04 mmol/L) and she was discharged on calcitriol 0.25 mg daily and caltrate, one tablet twice a day.

## Discussion

Graves’ disease is an autoimmune disorder that involves production of IgG antibodies that stimulate thyroid receptors present not only on the thyroid, but on adipocytes, fibroblasts, and osteons 2. Previously thought to be secondary to pituitary hyperfunction, these antibodies (TRAb) were first demonstrated in 1956 in sera from four patients: three with thyrotoxicosis and one with severe exophthalmos alone 3. Multiple hypotheses (molecular mimicry, bystander effect, and HLA typing) have proposed etiologies for the inciting trigger which, to date, remains unknown, but probably varies from patient to patient. This “hit” to the immune system results in autoantibody production and involves expression of multiple cytokines by T cells. Through a system of feedback loops, B cells are stimulated to become plasma cells and secrete stimulatory IgG antibodies. Interferon gamma (IFN‐*γ*), interleukin 2 (IL2), and tumor necrosis factor alpha (TNF‐*α*) are secreted by T‐helper 1 (TH1) cells, interleukin 4 and interleukin 5 are secreted by T‐helper 2 (TH2) cells, and interleukin 17 by T‐helper 17 (TH17) 2. These cytokines, particularly TNF‐*α* and IFN‐*γ*, are pro‐inflammatory and contribute to the inflammatory response in the systemic inflammatory response syndrome (SIRS). A subset of patients with Graves’ disease go onto develop thyroid storm, a severe hypercatabolic state with end‐organ complications. While this is most often a consequence of untreated hyperthyroidism, there is commonly an acute precipitating event such as infection, surgery, trauma, or parturition 2.

Bisphosphonates are commonly used to treat osteoporosis or acute symptomatic hypercalcemia. Intravenous bisphosphonates are nitrogenated and directly inhibit farnesyl diphosphate synthase. This enzyme is part of the mevalonate pathway (necessary for cell membrane anchoring, n‐glycosylation and steroid and hormone synthesis), causing depletion of geranylgeranyl pyrophosphate (GGPP)**,** reducing protein prenylation resulting in osteoclastic cell death. In‐vitro evidence has shown zoledronic acid not only causes *γδ* T‐cell expansion, facilitating tumor cell lysis through further cytokine release, but also facilitates C. pneumonia‐mediated immune responses 4. This causes gene and protein expression of TNF‐*α*, IL6, IL8, and IL12. GGPP depletion is implicated in the triggering of this cytokine response, with repletion of GGPP in‐vitro preventing acute zoledronic acid toxicity 5. Individuals with a greater pool of a particular subset of T cell, the gamma/delta T cell, have been identified to have a higher chance of developing an acute phase reaction upon phosphoantigen stimulation from bisphosphonate therapy 6. Interestingly, the gamma/delta T cell is elevated in the serum and present in the lymphocyte infiltration of the thyroid tissue in patients with Graves’ disease 7.

Considering the strong immune background in Graves’ disease, we hypothesize that in this patient, that current active Graves’ hyperthyroidism was a significant risk factor for the development of the acute phase reaction. In this case, we did not test for the individual cytokine levels but given the significant overlap of cytokines produced both in Graves’ disease and the acute phase reaction, we suspect the levels would have been difficult to be attributed to one condition over the other.

We recommend that, given the underlying autoimmune immunologic response already underway in patients with Graves’ thyrotoxicosis and hypercalcemia, bisphosphonate therapy should be avoided to avoid mimicking the SIRS and the potential risk of inciting an acute phase reaction. If bisphosphonate therapy is felt necessary, patients should be closely observed for the SIRS, and pre‐medication with paracetamol should be considered.

## Authorship

KG: involved in the patient's care and contributed to the conception, wrote the clinical record, wrote a quarter of the discussion, and approved the final version for publication. JE: involved in the patient's care and contributed to the conception, confirmed the accuracy of the clinical record, wrote majority of the discussion, and approved the final version for publication. RR: contributed to the conception, interpretation of the case report, revising the draft critically for important intellectual content, and approved the final version for publication. PS: involved in the patient's care and confirmed the accuracy of the clinical record, contributed to the conception, interpretation of the case report, revising the draft critically for important intellectual content, and approved the final version for publication. DS: contributed to the conception, interpretation of the case, revising the draft critically for important intellectual content, and approved the final version for publication.

## Conflict of Interest

None declared.
